# Interdisciplinary consensus statements on imaging of scapholunate joint instability

**DOI:** 10.1007/s00330-021-08073-8

**Published:** 2021-06-08

**Authors:** Tobias Johannes Dietrich, Andoni Paul Toms, Luis Cerezal, Patrick Omoumi, Robert Downey Boutin, Jan Fritz, Rainer Schmitt, Maryam Shahabpour, Fabio Becce, Anne Cotten, Alain Blum, Marco Zanetti, Eva Llopis, Maciej Bień, Radhesh Krishna Lalam, P. Diana Afonso, Vasco V. Mascarenhas, Reto Sutter, James Teh, Grzegorz Pracoń, Milko C. de Jonge, Jean-Luc Drapé, Marc Mespreuve, Alberto Bazzocchi, Guillaume Bierry, Danoob Dalili, Marc Garcia-Elias, Andrea Atzei, Gregory Ian Bain, Christophe L. Mathoulin, Francisco del Piñal, Luc Van Overstraeten, Robert M. Szabo, Emmanuel J. Camus, Riccardo Luchetti, Adrian Julian Chojnowski, Jörg G. Grünert, Piotr Czarnecki, Fernando Corella, Ladislav Nagy, Michiro Yamamoto, Igor O. Golubev, Jörg van Schoonhoven, Florian Goehtz, Maciej Klich, Iwona Sudoł-Szopińska

**Affiliations:** 1 Division of Radiology and Nuclear Medicine, Kantonsspital St. Gallen, Rorschacherstrasse 95, CH 9007 St. Gallen, Switzerland; 2 Faculty of Medicine, University of Zurich, Pestalozzistrasse 3, 8091 Zurich, Switzerland; 3 Norwich Medical School, University of East Anglia, Norwich Research Park, Norwich, NR4 7TJ UK; 4 Radiology Department, DMC-Diagnóstico Médico Cantabria, Castilla 6-Bajo, 39002 Santander, Spain; 5 Department of Diagnostic and Interventional Radiology, Lausanne University Hospital and University of Lausanne, Rue du Bugnon 46, 1011 Lausanne, Switzerland; 6 Department of Radiology, Stanford University School of Medicine, 300 Pasteur Drive, MC-5105, Stanford, CA 94305 USA; 7 Department of Radiology, New York University Grossman School of Medicine, NYU Langone Health, 660 First Avenue, New York, NY 10016 USA; 8 Klinikum der Ludwig-Maximilians-Universität München, Klinik und Poliklinik für Radiologie, Marchioninistraße 15, D-81377 München, Germany; 9 Department of Radiology, Universitair Ziekenhuis Brussel, Vrije Universiteit Brussel, Brussels, Belgium; 10 Service de Radiologie et Imagerie Musculosquelettique, CCIAL, CHU de Lille, 59800 Lille, France; 11 Guilloz Imaging Department, Central Hospital, University Hospital Center of Nancy, UDL, 29 avenue du Maréchal de Lattre de Tassigny, 54035 Nancy, France; 12 Department of Musculoskeletal Radiology, Clinic Hirslanden Zurich, Witellikerstrasse 40, 8008 Zurich, Switzerland; 13 Hospital de la Ribera, IMSKE, Valencia, Paseo Ciudadela 13, 46003 Valencia, Spain; 14 Gamma Medical Center, Broniewskiego 3, 01-785 Warsaw, Poland; 15 Department of Radiology, Robert Jones and Agnes Hunt Orthopaedic Hospital, Oswestry, UK; 16 Musculoskeletal Imaging Unit, Imaging Center, Radiology Department, Hospital da Luz, Grupo Luz Saúde, Av. Lusiada 100, 1500-650 Lisbon, Portugal; 17 Hospital Particular da Madeira, HPA, Funchal, Madeira Portugal; 18 AIRC, Advanced Imaging Research Consortium, Lisbon, Portugal; 19 Radiology, Balgrist University Hospital, University of Zurich, Forchstrasse 340, CH-8008 Zurich, Switzerland; 20 Department of Radiology, Nuffield Orthopaedic Centre, Oxford University Hospitals NHS Trust, Oxford, UK; 21 Department of Radiology, National Institute of Geriatrics, Rheumatology and Rehabilitation, Spartańska 1, 02-637 Warsaw, Poland; 22 Department of Radiology, St. Antonius Hospital Utrecht, Utrecht, The Netherlands; 23 Service de Radiologie B, Groupe Hospitalier Cochin, AP-HP Centre, Université de Paris, 75014 Paris, France; 24 Department of Medical Imaging, University Hospital Ghent, Corneel Heymanslaan 10, 9000 Ghent, Belgium; 25 Diagnostic and Interventional Radiology, IRCCS Istituto Ortopedico Rizzoli, Via G. C. Pupilli 1, 40136 Bologna, Italy; 26 MSK Imaging, University Hospital, 1 Avenue Molière, 67098 Strasbourg Cedex, France; 27 Epsom & St Helier University Hospitals NHS Trust Radiology Department, Dorking Road, Epsom, London, KT18 7EG UK; 28 Hand and Upper Extremity Surgery, Creu Blanca, P° Reina Elisenda 57, 08022 Barcelona, Spain; 29 Pro-Mano, Treviso, Italy and Ospedale Koelliker, Corso G. Ferraris 247, 10134 Torino, Italy; 30 Department of Orthopaedic Surgery, Flinders University, Bedford Park, Adelaide, South Australia Australia; 31 International Wrist Center, Clinique Bizet, 23 Rue Georges Bizet, 75116 Paris, France; 32 Instituto de Cirugía Plástica y de la Mano, Serrano 58 1B, 28001 Madrid, Spain; 33 Hand and Foot Surgery Unit (HFSU) SPRL, Rue Pierre Caille 9, 7500 Tournai, Belgium; 34 Department of Orthopaedics and Traumatology, Erasme University Hospital, Route de Lennik, 808 Brussels, Belgium; 35 Department of Orthopaedic Surgery, University of California Davis, Health System, 4800 Y Street, Sacramento, CA 95817 USA; 36 Hand Surgery Unit, Clinique de Lille Sud, 96 Rue Gustave Delory, Lesquin, France; 37 Rimini Hand Surgery and Rehabilitation Center, Rimini, Italy; 38 Orthopaedics and Trauma Department, Hand and Upper Limb Surgery, Norfolk and Norwich University NHS Trust Hospital, Colney Lane, Norwich, NR4 7UY UK; 39 Department of Hand, Plastic and Reconstructive Surgery, Kantonsspital St. Gallen, St. Gallen, Switzerland; 40 Traumatology, Orthopaedics and Hand Surgery Department, Poznan University of Medical Sciences, ul. 28 Czerwca 1956r. nr 135/147, 61-545 Poznań, Poland; 41 Orthopedic and Trauma Department, Hospital Universitario Infanta Leonor, C/ Gran Vía del Este N° 80, 28031 Madrid, Spain; 42 Hand Surgery Unit, Hospital Universitario Quirónsalud Madrid, Madrid, Spain; 43 Surgery Department, School of Medicine, Universidad Complutense de Madrid, Madrid, Spain; 44 Division for Hand Surgery and Surgery of Peripheral Nerves, Balgrist University Hospital, University of Zurich, Forchstrasse, 340, 8008 Zurich, Switzerland; 45 Department of Hand Surgery, Nagoya University, 65 Tsurumai-cho, Showa-ku, Nagoya, Japan; 46 Hand and Microsurgery Division, National Medical Research Centre of Traumatology and Orthopaedic named after N.N. Priorov, Moscow, Russia; 47 Clinic for Hand Surgery, Rhön Medical Center, Campus Bad Neustadt, Von Guttenberg-Straße 11, 97616 Bad Neustadt/Saale, Germany; 48 Department of Traumatology and Orthopaedics, Postgraduate Medical Center, A. Gruca Teaching Hospital, Otwock, Poland

**Keywords:** Wrist injuries, Joint instability, Diagnostic imaging, Guidelines, Surveys and questionnaires

## Abstract

**Objectives:**

The purpose of this agreement was to establish evidence-based consensus statements on imaging of scapholunate joint (SLJ) instability by an expert group using the Delphi technique.

**Methods:**

Nineteen hand surgeons developed a preliminary list of questions on SLJ instability. Radiologists created statements based on the literature and the authors’ clinical experience. Questions and statements were revised during three iterative Delphi rounds. Delphi panellists consisted of twenty-seven musculoskeletal radiologists. The panellists scored their degree of agreement to each statement on an eleven-item numeric scale. Scores of ‘0’, ‘5’ and ‘10’ reflected complete disagreement, indeterminate agreement and complete agreement, respectively. Group consensus was defined as a score of ‘8’ or higher for 80% or more of the panellists.

**Results:**

Ten of fifteen statements achieved group consensus in the second Delphi round. The remaining five statements achieved group consensus in the third Delphi round. It was agreed that dorsopalmar and lateral radiographs should be acquired as routine imaging work-up in patients with suspected SLJ instability. Radiographic stress views and dynamic fluoroscopy allow accurate diagnosis of dynamic SLJ instability. MR arthrography and CT arthrography are accurate for detecting scapholunate interosseous ligament tears and articular cartilage defects. Ultrasonography and MRI can delineate most extrinsic carpal ligaments, although validated scientific evidence on accurate differentiation between partially or completely torn or incompetent ligaments is not available.

**Conclusions:**

Delphi-based agreements suggest that standardized radiographs, radiographic stress views, dynamic fluoroscopy, MR arthrography and CT arthrography are the most useful and accurate imaging techniques for the work-up of SLJ instability.

**Key Points:**

• *Dorsopalmar and lateral wrist radiographs remain the basic imaging modality for routine imaging work-up in patients with suspected scapholunate joint instability*.

• *Radiographic stress views and dynamic fluoroscopy of the wrist allow accurate diagnosis of dynamic scapholunate joint instability*.

• *Wrist MR arthrography and CT arthrography are accurate for determination of scapholunate interosseous ligament tears and cartilage defects*.

## Introduction

Instability of the scapholunate joint (SLJ) is usually caused by insufficiency of the scapholunate interosseous ligament (SLIL) and secondary stabilizers [1]. A variety of static and dynamic diagnostic imaging techniques are being proposed for the work-up of SLJ instability, including radiography, fluoroscopy and ultrasonography, as well as computed tomography (CT) and magnetic resonance imaging (MRI), both with or without arthrography [1, 2]. Each imaging modality has its strengths and weaknesses. Although there is an increasing body of research in the literature, there are substantial uncertainties regarding the optimal diagnostic imaging work-up of wrist instability in clinical practice [2, 3].

Increasing interdisciplinary understanding and cooperation between radiologists and hand surgeons might help identify the most appropriate wrist instability imaging approach and ultimately optimize treatments and clinical outcomes. Therefore, a Delphi-based process was initiated by a small group of radiologists and hand surgeons to understand better the diagnostic performance of the various imaging techniques in wrist instability. The I-WRIST 2021 (*I*nternational *W*rist *R*adiologic evaluation for the *I*nstability of the *S*capholunate Joint and DRUJ/*T*FCC) group of radiologists and hand surgeons was established to provide interdisciplinary consensus statements on imaging of the two most frequent types of posttraumatic wrist instability that involves the SLJ and distal radioulnar joint (DRUJ)/triangular fibrocartilage complex (TFCC).

The purpose of this research was to establish evidence-based consensus statements on imaging of SLJ instability by experts using the Delphi technique for consensus-building.

## Materials and methods

### Panellists

The founders of the I-WRIST project (M.B., M.K., I.S.S.) invited experts in radiology and hand surgery for consensus-building. Twenty-seven radiologists with experience in clinical practice, research and teaching of musculoskeletal imaging from Switzerland (n = 5), France (n = 4), UK (n = 4), Poland (n = 3), Belgium (n = 2), Portugal (n = 2), Spain (n = 2), USA (n = 2), Germany (n = 1), Italy (n = 1) and the Netherlands (n = 1) were invited by the senior author (ISS). All radiologists consented to take part in the I-WRIST 2021 project. The senior author presented the consecutive stages of the I-WRIST project, particularly the Delphi technique for consensus-building and task leader of the scapholunate instability project (T.J.D.) during the first face-to-face meeting of the panellists at the annual meeting of the European Society of Musculoskeletal Radiology in Lisbon, Portugal, 2019 [4]. Hand surgeons recognized as experts in the diagnosis and management of wrist instabilities were invited by the I-WRIST founders to join the panel. Nineteen out of 22 hand surgeons from Spain (n = 3), France (n = 2), Germany (n = 2), Italy (n = 2), Poland (n = 2), Switzerland (n = 2), Australia (n = 1), Belgium (n = 1), Japan (n = 1), Russia (n = 1), UK (n = 1) and USA (n = 1) accepted the invitation.

### Questions

In the first step, hand surgeons were asked to develop questions on the imaging of SLJ instability to be put to the radiologists. The surgeons were also asked to select the most relevant clinical classifications to illustrate to radiologists the clinical relevance of imaging in the surgical decision-making process. A preliminary list of four SLJ instability questions was developed (Table 1). The selected classifications were the Garcia-Elias, Lluch and Stanley staging [5] (Table 2), the European Wrist Arthroscopy Society (EWAS) classification [6] (Table 3) and the Van Overstraeten and Camus classification [7] (Table 4).

**Table 1:** Preliminary list of four questions on scapholunate joint instability proposed by hand surgeons

No.	Question
1	Which imaging techniques can provide information on the type of lesions in the scapholunate joint instability according to Garcia-Elias staging (including cartilage lesions)?
2	Which imaging techniques can provide information on the type of scapholunate interosseous ligament lesion according to EWAS classification?
3	Which imaging techniques can provide information if the secondary stabilizers of the scapholunate joint, listed below, are intact or incompetent or completely torn? (RSCL, STTL, LRL, SRL, DRC, DIC)?
4	Which imaging techniques can provide information on the type of DCSS lesion according to Van Overstraeten and Camus classification?

Abbreviations: *DCSS* dorsal capsulo-scapholunate septum. *DIC* dorsal intercarpal ligament. *DRC* dorsal radiocarpal ligament. *EWAS* European Wrist Arthroscopy Society. *LRL* long radiolunate ligament. *RSCL* radioscaphocapitate ligament. *SRL* short radiolunate ligament. *STTL* scaphotrapezial-trapezoidal ligament

**Table 2:** Staging of scapholunate dissociations as proposed by Garcia-Elias et al [5]

Scapholunate dissociation stage	Anatomopathological abnormality
1	Is there a partial rupture with a normal dorsal scapholunate ligament?
2	If ruptured, can the dorsal scapholunate ligament be repaired?
3	Is the scaphoid normally aligned (radioscaphoid angle ≤ 45°)?
4	Is the carpal malalignment easily reducible?
5	Are the cartilages at both radiocarpal and midcarpal joints normal?
6	Complete scapholunate ligament injury with irreducible malalignment and cartilage degeneration?

**Table 3:** EWAS classification of scapholunate tears [6]

Arthroscopic stage (EWAS)	Arthroscopic testing of SLIL from midcarpal joint	Anatomopathological findings
I	No passage of the probe	Not found in the cadaver Specimens of Messina et al [6]
II lesion of membranous SLIL	Passage of the tip of the probe in the SL space without widening (stable)	Lesion of proximal/membranous part of SLIL
IIIA Partial lesion involving the palmar SLIL	Palmar widening on dynamic testing from MC joint (palmar laxity)	Lesion of palmar and proximal part of SLIL with or without lesion of RSCL- LRL
IIIB Partial lesion involving the dorsal SLIL	Dorsal SL widening on dynamic testing (dorsal laxity)	Lesion of proximal and dorsal part of SLIL with partial lesion of DIC
IIIC Complete SLIL tear, joint is reducible	Complete widening of SL space on dynamic testing, reducible with removal of probe	Complete lesion of SLIL (palmar, proximal, dorsal), complete lesion of one extrinsic ligament (DIC lesion or RSCL/ LRL)
IV Complete SLIL tear with SL gap	SL gap with passage of the arthroscope from MC to RC joint No radiographic abnormalities	Complete lesion of SLIL (palmar, proximal, dorsal), lesion of extrinsic ligaments (DIC and RSCL/ LRL)
V	Wide SL gap with passage of the arthroscope through SL joint. Frequent X Ray abnormalities such as an increased SL gap, DISI deformity	Complete lesion of SLIL, DIC, LRL, RSCL, involvement of one or more other ligaments (triquetrohamate, scaphotrapezial, DRC).

Abbreviations: *DIC* dorsal intercarpal ligament. *DISI* dorsal intercalated segmental instability. *DRC* dorso radiocarpal. *LRL* long radiolunate ligament. *MC* midcarpal. *RC* radiocarpal. *RSCL* radioscaphocapitate ligament. *SL* scapholunate. *SLIL* scapholunate interosseous ligament

**Table 4:** Classification of the dorsal capsulo-scapholunate septum as proposed by Van Overstraeten and Camus [7]

Stage	Arthroscopic findings
S0	Normal tension during palpation with a probe. Intact DCSS with continuous fibers mimicking cathedral arches
S1	DCSS loosened during palpation with a probe. Partial detached fibers with more than 50% continuous fibers
S2	DCSS elongated and loosened during palpation with a probe. Partial tear with less than 50% continuous fibers
S3	Totally torn DCSS or disappearance of DCSS

Abbreviation: *DCSS* dorsal capsulo-scapholunate septum

### Bibliographic search strategy

A word search of MEDLINE and the Cochrane Library using the terms ‘imaging’, ‘radiographs’, ‘magnetic resonance imaging’, ‘computed tomography’, ‘ultrasonography’, ‘scapholunate instability’, ‘scapholunate dissociation’, ‘scapholunate ligament tear’ and ‘scapholunate ligament injury’ revealed 696 articles. Subsequently, all radiologists were asked to supplement the literature database with additional publications on scapholunate instability. Publications were excluded if they were not meta-analyses or original scientific articles addressing techniques, diagnostic criteria and diagnostic performance data on imaging of SLJ instability. This left three meta-analyses and 91 original articles that formed the evidence base for the Delphi process and were archived in a cloud-based directory accessible to all radiologists.

### Task groups

The project leaders (T.J.D., I.S.S.) nominated experts into separate task groups. For consecutive Delphi rounds, each task group developed one statement as an answer to the assigned question in Table 1, followed by a short discussion and a list of references. The scientific evidence level according to the five-point scale developed by the Oxford Centre for Evidence-Based Medicine was assigned to every article of the discussion by the experts of each task group [8].

### Delphi process

Overall, three Delphi rounds were conducted using survey administration software (Google Forms, https://www.google.com/forms/about/). All 27 radiologists completed the first, second and third Delphi surveys in the periods 09/05/2020–05/06/2020, 12/07/2020–18/08/2020 and 27/09/2020–19/10/2020, respectively.

The panellists were asked to classify their degree of agreement to each statement according to an 11-point Likert-type scale, in which 0 reflects complete disagreement, 5 reflects neither agreement nor disagreement, greater than or equal to 8 (≥ 8) reflects agreement and 10 reflects complete agreement.

In the first Delphi round, the panellists were invited to comment on the phrasing or content of the four preliminary questions and statements listed in Table 1, particularly if their rating did not reflect full agreement (scoring ≤ 7). The feedback of the panellists was used to insert additional questions and rephrase the statements for the next Delphi rounds [4]. In this way, questions and statements underwent iterative revision according to the scores, suggestions and comments of the panellists of the first and second Delphi rounds in preparation for subsequent review. The second and third Delphi rounds included the revised and extended questions and statements and the corresponding questions and statements of the former round. Statistics and graphs illustrating the level of group agreement for each statement of the former round were included. Questions and statements #1–5 and #7 were added in the second Delphi round in response to panellists’ feedback to elaborate on the initial questions and statements. Statements were subdivided (#6.a–b, #8.a–d and #9.a–b) where the points were closely related, and new questions created when the points could be considered independently. The final fifteen questions and statements (#1–#10) of the second and third Delphi round are listed in Table 5. The third and final Delphi round was limited to the five questions and statements (#4, #5, #6.a, #6.b and #7) that did not achieve group consensus in the previous rounds. The end of the Delphi process was predetermined at a maximum of three rounds or achievement of group consensus for each statement, whichever came first [4].

**Table 5:** Second and third Delphi round: questions, statements and agreement of 27 panellists

No.	Questions and statements	Scientific evidence level *	Agreement [median] (IQR)
Which radiographs should be obtained for the diagnostic work-up of SLJ instability?			
#1	Dorsopalmar and lateral radiographs should be acquired as routine imaging work-up in patients with suspected SLJ instability. Radiographic stress views and dynamic fluoroscopy allow accurate diagnosis of dynamic SLJ instability.	3	89% (24/27) [9] (8–9)
Is MRI equivalent to MR arthrography (MRA) for the assessment of SLIL tears?			
#2	MRA provides better diagnostic accuracy for the determination of SLIL tears than MRI.	1	89% (24/27) [9] (9–10)
Is CT arthrography (CTA) appropriate for the assessment of SLIL tears?			
#3	CTA is very accurate for the determination of SLIL tears.	3	89% (24/27) [9] (9–10)
Should ultrasonography be included as part of the standard diagnostic work-up of SLJ instability?			
#4	Ultrasonography should not be part of the standard diagnostic work-up due to limited data on the diagnostic performance and reportedly low sensitivity.	3	100% (27/27) [9] (8–9)
Should kinematic-CT and kinematic-MRI be considered as standard imaging modalities for the SLJ instability?			
#5	Kinematic-CT and kinematic-MRI may detect dynamic SLJ instability; however, there are no established imaging protocols and guidelines for image interpretation outside dedicated imaging centers nor evidence showing an improved diagnostic accuracy of these techniques compared to dynamic fluoroscopy.	CT: 2 MRI: 3	96% (26/27) [9] (9–10)
Which imaging techniques can provide information if the secondary stabilizers of the scapholunate joint, listed below, are intact or incompetent or completely torn? (RSCL, LRL, SRL, STTL, DRC, DIC)?			
#6.a	Based on panellists’ expert opinion and a low scientific level of evidence, ultrasonography can delineate some extrinsic and intrinsic carpal ligaments, particularly the RSCL, LRL, DRC and DIC. However, validated scientific evidence on an accurate differentiation between partially or completely torn or incompetent ligaments is not available.	4	82% (22/27) [9] (8–10)
#6.b	Based on panellists’ expert opinion and a low scientific level of evidence, MRI/MRA can delineate most extrinsic and intrinsic carpal ligaments, particularly the RSCL, LRL, DRC and DIC. However, validated scientific evidence on an accurate differentiation between partially or completely torn or incompetent ligaments is not available. In contrast, some ligaments, such as the SRL and STTL, remain difficult to visualize.	4	93% (25/27) [9] (8–10)
Are CTA and MRA accurate for the assessment of cartilage defects in SLJ instability?			
#7	CTA and MRA are accurate for detecting cartilage defects; however, comparative data for imaging performance of the two modalities for assessing cartilage defects in SLJ instability are missing.	CTA: 3 MRA: 2	96% (26/27) [9] (9–10)
Which imaging techniques provide information on the type of lesions in SLJ instability according to Garcia-Elias staging system, including cartilage lesions?			
#8.a	Different imaging methods may provide accurate information for SLJ instability according to the Garcia-Elias staging, including a partial versus complete tear of the SLIL, quality of the dorsal scapholunate ligament, joint alignment and cartilage quality.	3	82% (22/27) [8] (8–9)
#8.b	Dorsopalmar and lateral radiographs as a basic imaging modality are generally recommended for the diagnostic work-up according to the Garcia-Elias staging, particularly for the evaluation of scaphoid alignment, advanced disease with complete scapholunate ligament injury, irreducible malalignment and cartilage degeneration (scapholunate dissociation stages 3 and 6).	3	100% (27/27) [9] (9–10)
#8.c	Stress radiographs combined with standard dorsopalmar/lateral radiographs or dynamic fluoroscopy enable evaluation on the reducibility of carpal malalignment (scapholunate dissociation stage 4).	3	93% (25/27) [9] (8–10)
#8.d	According to the Garcia-Elias staging, MRA or CTA are generally recommended for the diagnostic work-up for ligamentous and early cartilage defects (scapholunate dissociation stages 1 and 5). All four statements (#8.a–d) achieved group consensus in the second round. 82% (22/27), 100% (27/27), 93% (25/27) and 100% (27/27) of the panel rated the items ‘8’ or higher).	CTA: 3 MRA: 3	100% (27/27) [9] (9–10)
Which imaging techniques provide information on the type of SLIL lesion according to the EWAS classification?			
#9.a	CTA and MRA supplemented by dynamic studies, if essential, provide the most accurate diagnosis of proximal, palmar and dorsal lesions of the SLIL and partial and complete tears of the secondary stabilizers, according to the EWAS classification.	CTA: 3 MRA: 3	96% (26/27) [9] (8–10)
#9.b	Dorsopalmar and lateral radiographs are generally recommended as an initial imaging modality for the diagnostic work-up according to the EWAS classification, but their specificity is limited to advanced stages, such as increased scapholunate gap and DISI deformity.	3	96% (26/27) [9] (9–10)
Which imaging techniques can accurately diagnose the type of DCSS lesion, according to the Van Overstraeten and Camus classification [8]?			
#10	Based on panellists’ expert opinion, MRA and CTA provide the most accurate diagnosis of DCSS tears, although scientific evidence is not available.	5 (expert opinion)	82% (22/27) [9] (8–10)

Abbreviations: *CTA* CT arthrography. *DCSS* dorsal capsulo-scapholunate septum. *DIC* dorsal intercarpal ligament. *DISI* dorsal intercalated segmental instability. *DRC* dorsal radiocarpal ligament. *EWAS* European Wrist Arthroscopy Society. *IQR* Interquartile Range. *LRL* long radiolunate ligament. *MRA* MR arthrography. *MRI* magnetic resonance imaging. *RSCL* radioscaphocapitate ligament. *SLJ* scapholunate joint. *SLIL* scapholunate interosseous ligament. *SRL* short radiolunate ligament. *STTL* scaphotrapezial-trapezoidal ligament. Asterisk (*) indicates scientific evidence level according to the five-item scale of the Oxford Centre for Evidence-Based Medicine [8]

### Statistical analysis

Standards for consensus in Delphi surveys have never been established [9]. A systematic review revealed that the median threshold for consensus of Delphi studies was 75% with a broad range between 50 and 97% [10]. Group consensus of the present survey was defined as 80% or more of the panellists rating their agreement level as ‘8’, ’9’ or ‘10’ [11]. Median and interquartile range (IQR) values are provided as supplemental measures of polarization among the panellists [9].

## Results

Literature research on diagnostic imaging of SLJ instability revealed a heterogeneous spread of scientific evidence between level 1 and level 5 according to criteria of the Oxford Centre for Evidence-Based Medicine (Table 5) [8]. Ten statements achieved group consensus in the second Delphi round (statements #1, #2, #3, #8.a–d, #9.a–b, #10). The remaining five statements achieved group consensus in the third Delphi round (statements #4, #5, #6.a–b and #7). The percentages, medians and IQRs of agreement of the Delphi panellists are provided in Table 5.

The final questions and statements are listed below:

- *Question #1*: Which radiographs should be obtained for the diagnostic work-up of SLJ instability?
- *Statement #1*: Dorsopalmar and lateral radiographs should be acquired as routine imaging work-up in patients with suspected SLJ instability. Radiographic stress views and dynamic fluoroscopy allow accurate diagnosis of dynamic SLJ instability.

Statement #1 achieved group consensus in the second round. Eighty-nine percent (24/27) of the panel rated the items ‘8’, ’9’ or ‘10’.

- *Question #2*: Is MRI equivalent to MR arthrography (MRA) for the assessment of SLIL tears?
- *Statement #2*: MRA provides better diagnostic accuracy for the determination of SLIL tears than MRI.

Statement #2 achieved group consensus in the second round. Eighty-nine percent (24/27) of the panel rated the items ‘8’ or higher.

- *Question #3*: Is CT arthrography (CTA) appropriate for the assessment of SLIL tears?
- *Statement #3*: CTA is very accurate for the determination of SLIL tears.

Statement #3 achieved group consensus in the second round. Eighty-nine percent (24/27) of the panel rated the items ‘8’ or higher.

- *Question #4*: Should ultrasonography be included as part of the standard diagnostic work-up of SLJ instability?
- *Statement #4*: Ultrasonography should not be part of the standard diagnostic work-up due to limited data on the diagnostic performance and reportedly low sensitivity.

Statement #4 achieved group consensus in the third round. One hundred percent (27/27) of the panel rated the items ‘8’ or higher.

- *Question #5*: Should kinematic-CT and kinematic-MRI be considered as standard imaging modalities for the SLJ instability?
- *Statement #5*: Kinematic-CT and kinematic-MRI may detect dynamic SLJ instability; however, there are no established imaging protocols and guidelines for image interpretation outside dedicated imaging centers nor evidence showing an improved diagnostic accuracy of these techniques compared to dynamic fluoroscopy.

Statement #5 achieved group consensus in the third round. Ninety-six percent (26/27) of the panel rated the items ‘8’ or higher.

- *Question #6*: Which imaging techniques can provide information if the secondary stabilizers of the scapholunate joint, listed below, are intact or incompetent or completely torn?
  - Radioscaphocapitate ligament (RSCL)
  - Long radiolunate ligament (LRL)
  - Short radiolunate ligament (SRL)
  - Scaphotrapezial-trapezoidal ligament (STTL)
  - Dorsal radiocarpal ligament (DRC)
  - Dorsal intercarpal ligament (DIC)
- *Statement #6.a*: Based on panellists’ expert opinion and a low scientific level of evidence, ultrasonography can delineate some extrinsic and intrinsic carpal ligaments, particularly the RSCL, LRL, DRC and DIC. However, validated scientific evidence on an accurate differentiation between partially or completely torn or incompetent ligaments is not available.
- *Statement #6.b*: Based on panellists’ expert opinion and a low scientific level of evidence, MRI/MRA can delineate most extrinsic and intrinsic carpal ligaments, particularly the RSCL, LRL, DRC and DIC. However, validated scientific evidence on an accurate differentiation between partially or completely torn or incompetent ligaments is not available. In contrast, some ligaments, such as the SRL and STTL, remain difficult to visualize.

Both statements #6.a and #6.b achieved group consensus in the third round. Eighty-two percent (22/27) and 93% (25/27) of the panel rated the items ‘8’ or higher.

- *Question #7*: Are CTA and MRA accurate for the assessment of cartilage defects in SLJ instability?
- *Statement #7*: CTA and MRA are accurate for detecting cartilage defects; however, comparative data for imaging performance of the two modalities for assessing cartilage defects in SLJ instability are missing.

Statement #7 achieved group consensus in the third round. Ninety-six (26/27) of the panel rated the items ‘8’ or higher.

- *Question #8*: Which imaging techniques provide information on the type of lesions in SLJ instability according to Garcia-Elias staging system, including cartilage lesions?
- *Statement #8.a*: Different imaging methods may provide accurate information for SLJ instability according to the Garcia-Elias staging, including a partial versus complete tear of the SLIL, quality of the dorsal scapholunate ligament, joint alignment and cartilage quality.
- *Statement #8.b*: Dorsopalmar and lateral radiographs as a basic imaging modality are generally recommended for the diagnostic work-up according to the Garcia-Elias staging, particularly for the evaluation of scaphoid alignment, advanced disease with complete scapholunate ligament injury, irreducible malalignment and cartilage degeneration (scapholunate dissociation stages 3 and 6).
- *Statement #8.c*: Stress radiographs combined with standard dorsopalmar/lateral radiographs or dynamic fluoroscopy enable evaluation on the reducibility of carpal malalignment (scapholunate dissociation stage 4).
- *Statement #8.d*: According to the Garcia-Elias staging, MRA or CTA are generally recommended for the diagnostic work-up for ligamentous and early cartilage defects (scapholunate dissociation stages 1 and 5).

All four statements (#8.a–d) achieved group consensus in the second round. Eighty-two percent (22/27), 100% (27/27), 93% (25/27) and 100% (27/27) of the panel rated the items ‘8’ or higher.

- *Question #9*: Which imaging techniques provide information on the type of SLIL lesion according to the EWAS classification?
- *Statement #9.a*: CTA and MRA supplemented by dynamic studies, if essential, provide the most accurate diagnosis of proximal, palmar and dorsal lesions of the SLIL and partial and complete tears of the secondary stabilizers, according to the EWAS classification.
- *Statement #9.b*: Dorsopalmar and lateral radiographs are generally recommended as an initial imaging modality for the diagnostic work-up according to the EWAS classification, but their specificity is limited to advanced stages, such as increased scapholunate gap and DISI deformity.

Both statements #9.a and #9.b achieved group consensus in the second round. Ninety-six percent (26/27) and 96% (26/27) of the panel rated the items ‘8’ or higher.

- *Question #10*: Which imaging techniques can accurately diagnose the type of DCSS lesion, according to the Van Overstraeten and Camus classification?
- *Statement #10*: Based on panellists’ expert opinion, MRA and CTA provide the most accurate diagnosis of DCSS tears, although scientific evidence is not available.

Statement #10 achieved group consensus in the second round. Eighty-two percent (22/27) of the panel rated the items ‘8’ or higher.

## Discussion

The most important findings of this consensus agreement are that radiographs, radiographic stress views, dynamic fluoroscopy, MRA and CTA are either the most accurate imaging techniques for the diagnostic work-up of SLJ instability or, in the absence of published scientific evidence, considered to be the most reasonable by the I-WRIST expert panel.

**Fig. 1:**
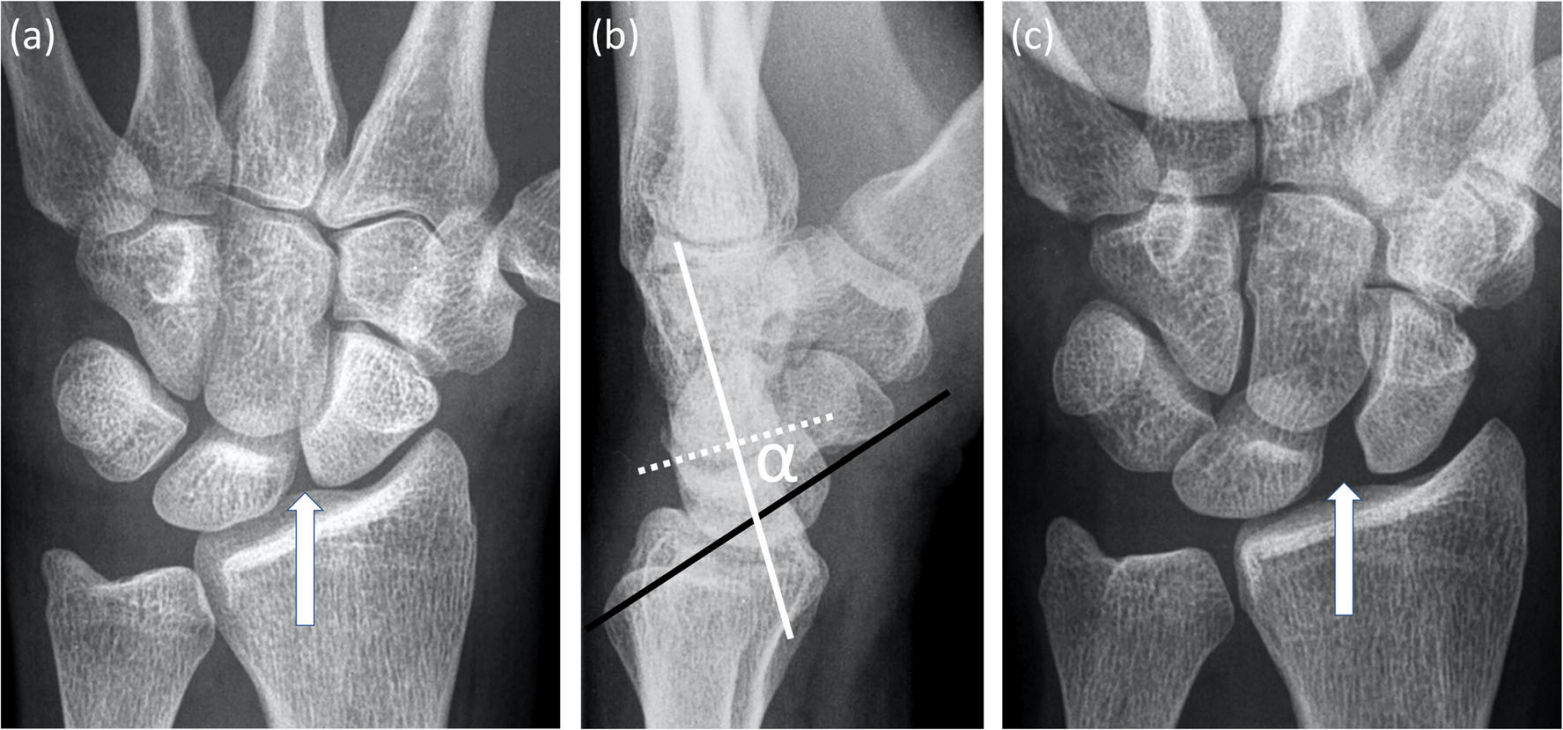
A 28-year-old male patient with symptoms of scapholunate joint instability after a left-sided rotational wrist injury due to accidentally jammed drilling machine. **a** Dorsopalmar radiograph shows a slightly increased scapholunate distance (arrow) and a signet ring sign of the scaphoid. **b** The lateral radiograph presents an abnormally increased scapholunate angle of 73° (α). **c** Dorsopalmar clenched ball view as a radiographic stress view demonstrates a definitely abnormal increased scapholunate distance

Standardized dorsopalmar and lateral radiographs remain the first-line imaging approach for assessing patients with SLJ instability where malalignment of the carpal bones may indicate static instability [12–19] (Fig. 1)

.

The large number of 27 panellists from eleven different countries including the USA and Europe was selected to ensure that the consensus statements were based on broad expert opinions from a heterogeneous clinical background. This was reflected in the first round of the Delphi process where it proved a challenge to gain consensus on the preliminary list of four questions on scapholunate joint instability proposed by the hand surgeons (Table 1). These four questions were redrafted based on the scores and comments obtained during the first Delphi round to create more focused questions with rephrased and subdivided statements that presented more specific statements about the utility of the various imaging techniques for the next Delphi rounds [4]. This iterative revision of questions and statements achieved group consensus for ten statements in the second Delphi round and for five statements in the third Delphi round.

Question and statements addressing ultrasonography, kinematic-CT, kinematic-MRI and assessment of extrinsic carpal ligaments underwent a third Delphi round. This may be because the opinions and experience of the individual panellist for these topics are more heterogeneous than for those questions and statements that achieved consensus in round 2. The topics covered in the third Delphi round were also characterized by either low levels of scientific evidence and small numbers of published original articles.

Radiographic stress views such as variations of clenched fist views allow detection of dynamic SLJ instability in patients with a normal scapholunate distance on standardized dorsopalmar radiographs in neutral position [12].

Dynamic fluoroscopy provides real-time interactive imaging to differentiate between static and dynamic SLJ instability [14, 15, 20]. A sensitivity of 90%, a specificity of 97% and a diagnostic accuracy of 93% were reported for dynamic fluoroscopy in detecting SLJ instability [20].

MRI and MRA demonstrate high diagnostic accuracy for detecting SLIL tears [21–30], with MRA outperforming conventional MRI [21]. A meta-analysis for detecting SLIL injury determined a sensitivity of 82% and specificity of 93% for MRA compared to surgery or gross pathology as standard of reference [24].

**Fig. 2:**
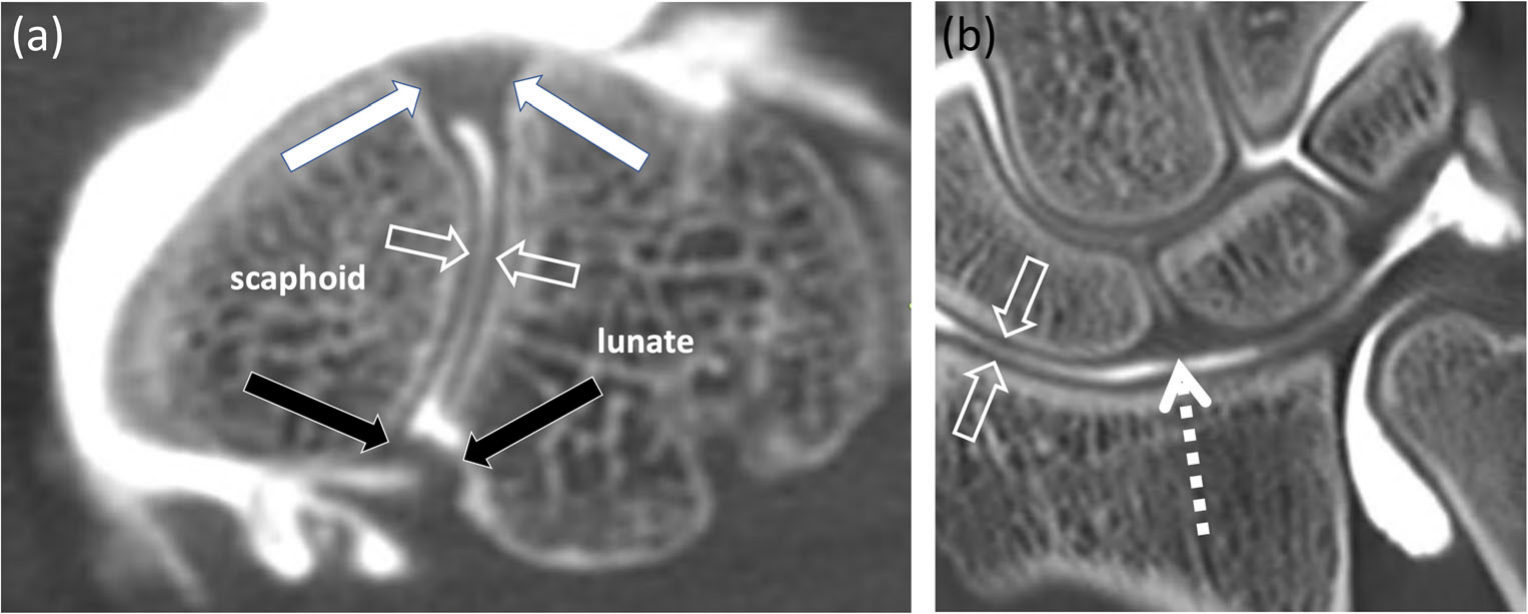
A 20-year-old male patient underwent tricompartmental CT arthrography to assess the scapholunate ligaments after a wrist trauma. CT arthrography demonstrates normal findings with continuity of the palmar band (**a**, black arrows), the dorsal band (**a**, white arrows) and proximal/membranous band (**b**, dashed arrow) of the scapholunate ligament on transverse (**a**) and coronal images (**b**). Open arrows (**a**, **b**) indicate regular articular cartilage in both imaging planes

CTA demonstrates high diagnostic accuracy for detecting SLIL tears [21–23, 31–33] (Fig. 2)

, which is higher than conventional MRI [21]. A sensitivity of 94% and specificity of 86% were reported to detect SLIL tears on CTA compared to arthroscopy as standard of reference [32].

A very high specificity (100%) and high diagnostic accuracy (89%) but a low sensitivity (< 50%) were reported for ultrasonography in the diagnostic work-up of SLJ instability [34].

Kinematic-CT can diagnose SLJ instability, especially when the conventional diagnostic evaluation is inconclusive [35–38]. It has been suggested that kinematic-MRI is a fast and reliable technique for detecting dynamic SLJ instability with a diagnostic accuracy comparable to dynamic fluoroscopy [39–41]. Aside from the lack of established protocols, implementation of kinematic cross-sectional imaging requires a change in workflow and training of the medical staff. Although promising, the applicability of kinematic-CT and kinematic-MRI in clinical routine may be challenging. MRI, MRA and ultrasonography can identify several intact or torn secondary stabilizers of the SLJ but the level of evidence for the accuracy of these tests is low due to a small number of study participants and importantly a lack of valid reference standards [42–46]. The assessment of secondary stabilizers of the SLJ on ultrasonography demands an experienced and subspecialized examiner, which may further limit its applicability in many institutions.

Advanced osteoarthritis with articular cartilage defects can be diagnosed by conventional radiography, but CTA and MRA are considered to be more accurate for diagnosing early cartilage defects. CTA has a reported sensitivity of between 45 and 100% and a specificity of 93 to 100% for detecting wrist cartilage defects [22, 32, 33], whereas MRA has a sensitivity of 84% and a specificity of 96% [30].

The Garcia-Elias staging of SLIL injuries focuses on static and dynamic evaluation of the SLJ and the SLIL [6] (Table 2). This 6-stage scapholunate dissociation grading system evaluates SLJ dysfunction from the least to most advanced stages, including differentiation between complete and partial SLIL rupture with a normal dorsal scapholunate ligament, evaluation of SLJ alignment and reducibility and assessment of radiocarpal and midcarpal articular cartilage.

Dynamic evaluation of SLJ instability, originally proposed as a radiographic criterion in the Garcia-Elias staging (scapholunate dissociation stage 4), may also be evaluated by dynamic fluoroscopy, ultrasonography, kinematic-CT and kinematic-MRI. As of yet, there is no comparative data for determining the accuracy of these dynamic modalities.

The EWAS classification [7] (Table 3) requires static and dynamic arthroscopic evaluation of the SLJ to assess the anatomopathological structures, particularly the SLIL and several secondary stabilizers of the SLJ. The scientific literature demonstrates that CTA or MRA alone provides the most accurate morphological assessment of the most anatomopathological structures involved in the EWAS classification; however, some ligaments are not consistently visible on MRI or MRA, such as the triquetrohamate and scaphotrapezial ligaments [1, 42, 43, 46].

A limitation of this work relates to the selection of the expert panel members. Most of the radiologists came from university teaching hospitals and had an academic track record in musculoskeletal imaging and were selected predominantly from Europe. Therefore, the constitution of the expert panel may bias the results against the practical and theoretical perspective of non-academic radiologists, particularly outside Europe.

In conclusion, the present consensus agreement suggests that radiographs, radiographic stress views, dynamic fluoroscopy, MRA and CTA are currently the most useful and accurate imaging techniques for the work-up of SLJ instability.

## Abbreviations

CT: Computed tomography
CTA: Computed tomography arthrography
DCSS: Dorsal capsulo-scapholunate septum
DIC: Dorsal intercarpal ligament
DISI: Dorsal intercalated segmental instability
DRC: Dorsal radiocarpal ligament
DRUJ: Distal radioulnar joint
EWAS: European Wrist Arthroscopy Society
IQR: Interquartile range
I-WRIST 2021: International Wrist Radiologic evaluation for the Instability of the Scapholunate and DRUJ/TFCC
LRL: Long radiolunate ligament
MC: Midcarpal
MRA: Magnetic resonance arthrography
MRI: Magnetic resonance imaging
OA: Osteoarthritis
RC: Radiocarpal
RSCL: Radioscaphocapitate ligament
SL: Scapholunate
SLIL: Scapholunate interosseous ligament
SLJ: Scapholunate joint
SRL: Short radiolunate ligament
STTL: Scaphotrapezial-trapezoidal ligament
TFCC: Triangular fibrocartilage complex

## Declarations

### Guarantor

The scientific guarantor of this publication is Tobias Johannes Dietrich (first author) and Iwona Sudoł-Szopińska (senior author).

### Statistics and Biometry

Małgorzata Mańczak kindly provided statistical advice for this manuscript.

### Methodology

• Prospective

• Multicentre study
